# Habits as learning enhancers

**DOI:** 10.3389/fnhum.2014.00918

**Published:** 2014-11-14

**Authors:** Gloria Balderas

**Affiliations:** Departamento de Filosofía, Universidad de NavarraPamplona, Spain

**Keywords:** habit, hierarchical model, action sequence, fast mapping, exclusion learning

## Introduction

Habits are usually associated with both a positive and a negative consequence. The positive consequence is that habits liberate attentional resources and mechanisms (James, 1984, p. 129), thus enabling organisms to perform simultaneous or more complex actions. The negative consequence is that habits become rigid behaviors which persist despite producing harmful outcomes, as in addictions and some neurological disorders. This article proposes that habits also function as learning enhancers. The plausibility of this statement is supported by results from research on word-trained dogs. The use of an animal example has the advantage of parsimony, since it makes possible to show the capacity of habits to facilitate new learning without appealing to highly sophisticated human competences.

Evidence has been found that dogs are able to *fast map* (Kaminski et al., 2004). In studies of language acquisition, the ability to make accurate assumptions about the referent of an unfamiliar word is called *fast mapping*, a phenomenon that has been observed especially in toddlers (Carey and Bartlett, 1978; Swingley, 2010). This article argues that the training in words forms habits that predispose dogs to establish a new word-object association.

The definition of learning as ontogenetic adaptation (De Houwer et al., 2013, p. 633) and the hierarchical view of habit (Dezfouli and Balleine, 2012, 2013) are expounded in Section Learning and Habits. Taking into account these notions, the results of experiments on *fast mapping* in dogs are presented in Section Fast Mapping in Dogs and Learned Habits, to show that habits work as learning enhancers. Finally, there is a brief section of Concluding Remarks.

## Learning and habits

This section presents a functional definition of learning and the hierarchical view of habits. These two notions serve as a framework to present the results on fast mapping in dogs. Learnings defined as ontogenetic adaptation are “changes in the behavior of an organism that are the result of regularities in the environment of that organism” (De Houwer et al., 2013, p. 633). This definition applies to any observable behavior of living organisms, provided that this behavior is a response to stimuli in their (past or present) environment.

The relevant regularities for learning are stimuli or behaviors that are repeated over time or that are present more than one at a time. Importantly, since this definition includes the behavior of the organism itself as a regularity, it can favor the claim that habits are learning enhancers.

Causality between regularities and behavior is functional: the regularity in the environment can be described as an independent variable whose properties determine the behavior (the dependent variable). In this sense, to say that an organism has learned something is equivalent to a hypothesis about how a (past or present) regularity has caused a change in behavior. Moreover, the definition means that learning is only an adaptation—because it occurs due to a regularity—but not that it must be adaptive (advantageous for the organism).

Dezfouli and Balleine (2012, 2013) have presented evidence for the hierarchical view of habits. In their proposal, habits are more complex than goal-directed actions. Habits are action sequences—macro actions composed of primitive actions—under the control of a global goal-directed system that also governs goal-directed actions. In virtue of this system, the organism can opt for simple (goal-directed) actions or launch a sequence to achieve its goals in efficient manner.

To explain how these sequences are consolidated, Dezfouli and Balleine distinguish between *closed-loop* and *open-loop* execution. At the beginning of learning, feedback is crucial. The organism needs a reward or some clues in the environment to identify and perform the proper behavior (*closed-loop execution*). In advanced stages of training, a step in the sequence is conditioned by the previous step, regardless of feedback stimuli or reward (*open-loop execution*). This independence accounts for the insensitivity to the outcome shown in experiments of reward devaluation and contingency degradation that are standard measures to determine if a habit has been acquired (Dickinson et al., 1983; Dickinson, 1985).

When a sequence is consolidated, reaction times decrease. This occurs because the organism is not evaluating the reward for each primitive action but is acting based on an average of the reward received by previous executions of the macro action. If the environment changes and habitual behavior becomes maladaptive, the sequence can disintegrate after some time when the average reward is diminished.

Since the sequence constitutes a unit and the steps are interdependent, the organism tends to complete it. Each primitive action performed functions as a signal to execute the next action. However, the goal-directed system can regain control to facilitate the learning of new sequences (Dezfouli and Balleine, 2012, pp. 1047–1048; 2013, pp. 10–11).

In addition, the hierarchical view predicts that if the organism must make a decision in the initial state of a sequence, it exhibits habitual behavior; but if the decision point coincides with a mid-state sequence, the behavior will be goal-directed.

## Fast mapping in dogs and learned habits

A purpose of training dogs with words is to elucidate whether other species share some of the mechanisms involved in human language. Typically, the association between a label and an item is done by presenting simultaneously the object and its name; then the dog is allowed to explore or play with the object; finally, the animal is requested to deliver the item and is rewarded if its behavior is correct.

Several studies have confirmed that some dogs are able to relate an unfamiliar word with a new object, an ability similar to human *fast mapping*.

Kaminski et al. (2004) have examined Rico, a dog that has learned over 200 label-item associations. First, the performance of Rico was tested with a simple version of the fetching-game: the dog was asked to bring a familiar item from another room. Rico correctly brought 37 items during 40 trials. Second, *fast mapping* was tested in sessions in which an unknown item was placed among 7 familiar items: after requesting for one or two familiar items, a new word was used to ask Rico to bring an item. Rico brought the new item in 7 of 10 sessions.

The researchers assumed that Rico's performance could include, among others, a general mechanism for exclusion learning. Markman and Abelev (2004) have suggested that Rico could choose the correct item due to a bias toward novelty; but this objection has been refuted by showing that dogs are able to ignore new items (Fischer et al., 2004; Aust et al., 2008; Pilley and Reid, 2011; Grassmann et al., 2012). In what follows it is assumed that dogs are capable of learning by exclusion. I will attempt to show that this learning is supported by the acquisition of two habits, described according to the hierarchical view: the item-label association and the fetching-game.

At first glance, it seems that the label-item association does not constitute a sequence; however it is a complex behavior. Even in the absence of distracting items, it is possible to distinguish three primitive actions: (i) *search*; (ii) *match*; and (iii) *approximation*. The dog has to look for the object (i); match the item with its label (ii) when the correct item is in view; and show some other behavior (iii) indicating that recognition (i.e., take, paw). It could be insisted that the association is a simple behavior because is identified with the matching (a cognitive response); but in the experimental context, this response is accessible to the observer only because is preceded by the search and followed by another action. Therefore, the execution of the association task must also include these steps. When the macro action has been acquired, animals run it fast.

The fetching-game is a sequence separable from the association task. There is evidence that dogs are able to combine different types of orders with various label-item pairs, therefore dogs can learn different games with the same objects (i.e., pointing-game) (Pilley and Reid, 2011; Ramos and Ades, 2012).

The *fetching-game* is the main macro action which includes *selecting the correct item* as a subordinate macro action. The fetching-game consists in going for and delivering the requested item. Primitive actions begin when Rico receives the order to bring the object. Rico executes three main tasks: (a) go for; (b) select; and (c) deliver; *b* is in turn divided in *look-for, match*, and *take*. It should be added that since each matching is different, the animal acquires new sequences every time it learns a word; so that each label-item pair increases its resources for an efficient performance.

When a familiar item is requested *a, b*, and *c* function as a unit and the steps are executed without interruption. In the experiments of fast mapping, this behavior changes, and this change can reveal the role of habits in both the detection and the solution of a problem.

### Detecting the problem

The dog immediately executes *a* (go for) in response to the request with the unknown word, but the execution stops at *b* (select). This suggests that Rico detects a problem. Rico executes *look-for* but is stuck in *match*, therefore it can not *take* (see also Pilley and Reid, 2011, p. 193). This behavior fits the hierarchical view, in which each primitive action within a sequence is a signal to execute the next action. In this case, *take* can not start because *match* was not executed. Since Rico does not dispose of a name-object association that enables it to complete the task, it is in a situation where it has to make a decision in the middle of the selection task, so the goal-directed system regains control. After solving the problem, the fetching-game sequence follows its tendency to completion and Rico returns to the sequence: it goes to *take* and to *c* (deliver). This description also follows the hierarchical view because at the starting point the behavior begins as a habit, when a decision is required it becomes goal-directed and ends again as a habit after overcoming the difficulty.

### Solving the problem

Successful selection of new items is explained (in part) because dogs use the exclusion mechanism to eliminate options. This process involves the goal-directed system. Nevertheless, exclusion requires a criterion to determine which items must be excluded. The key point is that this criterion is provided by the association sequences consolidated during training. The learned label-item pairs prevent the animal from matching previously labeled items with new labels, and therefore, they guide it to match the new sound with the unnamed item. In addition, the context of the fetching-game also models the behavior of the animal since in this main macro action the reward depends on delivering a specific (correct) item which forces the animal to choose one item rather than perform any other action.

## Concluding remarks

Exclusion learning involved in fast mapping can be described according to the hierarchical view of habits. To the extent that habits are consolidated sequences, they can be considered as a type of behavioral regularity. According to the definition of learning as ontogenetic adaptation, behavioral regularities can lead to learning. The performance of Rico and other dogs manifest how habits modulate behavior and guide the animal to detect and solve a problem.

The problem is the absence of a label-item pair that allows completion of the sequences of association and fetching-game. The typical response of word-trained dogs is to stop acting: they do not choose any of the known items. In this situation the role of the habit as learning enhancer resides in the dynamism of the sequence that tends to be completed. If this dynamism is interrupted, the goal-directed system starts the exclusion process.

In addition, overcoming the problem requires habits in two ways. First, the overall context of game-fetching constrains the behavior of the animal to choose only one item. Moreover, the set of available association sequences provides the criteria that eliminate all familiar items that already have a name.

Thus, in this example of behavioral research on animals, acquired habits can be seen as regularities that lead to new learning. This case shows the plausibility of habits as learning enhancers in a parsimonious way. If this claim is accepted, the possibility of a new line of research is open.

### Conflict of interest statement

The author declares that the research was conducted in the absence of any commercial or financial relationships that could be construed as a potential conflict of interest.
